# A Pilot Study of MicroRNAs Expression Profile in Serum and HBsAg Particles: Predictors of Therapeutic Vaccine Efficacy in Chronic Hepatitis B Patients: Erratum

**DOI:** 10.1097/01.md.0000483157.71616.d9

**Published:** 2016-05-06

In the article “A Pilot Study of MicroRNAs Expression Profile in Serum and HBsAg Particles: Predictors of Therapeutic Vaccine Efficacy in Chronic Hepatitis B Patients”,^1^ which appeared in Volume 95, Issue 2 of *Medicine*, several typos appeared. The article has since been corrected
